# Patient Visits and Prescribing Patterns Associated with Rosacea in Korea: A Real-World Retrospective Study Based on Electronic Medical Records

**DOI:** 10.3390/jcm11061671

**Published:** 2022-03-17

**Authors:** Yu Ri Woo, Hyun Jeong Ju, Jung Min Bae, Minah Cho, Sang Hyun Cho, Hei Sung Kim

**Affiliations:** 1 Department of Dermatology, Incheon St. Mary’s Hospital, The Catholic University of Korea, Seoul 06591, Korea; w1206@naver.com (Y.R.W.); macho_maria@naver.com (M.C.); drchos@yahoo.co.kr (S.H.C.); 2 Department of Dermatology, St. Vincent’s Hospital, The Catholic University of Korea, Seoul 06591, Korea; hyd0116@naver.com; 3 Heal House Skin Clinic, Mesanro 24, Paldal-gu, Suwon 16461, Korea; jminbae@gmail.com

**Keywords:** rosacea, visits, prescription, Korea, trends

## Abstract

Rosacea is a common and chronic inflammatory skin disorder. The visiting trends and prescribing patterns involving Korean patients with rosacea have not been thoroughly examined. To examine the visiting trends of patients with rosacea, and to analyze the prescription patterns of Korean dermatologists managing such patients, a retrospective cross-sectional study on dermatology outpatients who visited the seven affiliated hospitals of The Catholic University of Korea between 2007 and 2018 was performed. A total of 56,651 visits were made by rosacea patients. The mean annual number of hospital visits made by rosacea patients over a 6-year period increased from 2456 in 2007–2012 to 6985 in 2013–2018. Hospital visits were most prevalent in female patients aged 40 to 59 years. There was no statistically significant difference in patient visitation between the seasons. As for prescriptions, systemic antibiotics were most commonly prescribed, followed by antihistamines, non-steroidal anti-inflammatory drugs, and retinoids. Among the topical agents, metronidazole was the most prescribed agent during 2007–2012, whereas calcineurin inhibitors were favored most during 2013–2018. Dermatology outpatient visits by individuals with rosacea have increased in Korea over time. The real-world prescription trend presented here may help dermatologists facilitate better treatment strategies and provide appropriate guidance to patients with rosacea.

## 1. Introduction

Rosacea is a common chronic inflammatory skin disorder of multifactorial etiology. It is characterized by cutaneous signs such as fixed centrofacial erythema, phymatous changes, flushing, papulopustules, and telangiectasia [1]. The worldwide prevalence of rosacea is estimated to be 5.46% of the adult population [2]. The prevalence of rosacea is relatively high in fair-skinned individuals, such as those of Celtic and North European descent [3], but it is also seen in Africans and Asians [4,5,6]. The prevalence of rosacea does vary according to ethnicity, suggesting the need to elucidate its prevalence in Koreans. Poor knowledge and a lack of awareness of rosacea may contribute to the underdiagnosis of rosacea in Asians, which needs to be corrected. With rapid urbanization and industrialization over the last decade, there is a growing interest in facial dermatoses such as rosacea. However, the trends in patients’ visits and the prescribing patterns of Korean dermatologists for rosacea are yet to be investigated.

Owing to its multifactorial etiology, the management of rosacea requires a combined approach. Appropriate skin care, sun protection, and the avoidance of possible triggering factors should be a part of one’s daily routine. In addition to these lifestyle modifications, a number of pharmacological and laser therapies can be used either singly or in combination to manage rosacea. Recently, expert panels have proposed updated treatment guidelines for rosacea [7,8]. However, it is unclear whether the real-world treatment pattern has changed accordingly, since there may be restrictions or limited access to the novel treatment options introduced in the updated guidelines.

To date, little is known about the visiting trends of Korean patients with rosacea. Moreover, the current real-world practice pattern of rosacea is not yet reported. Therefore, the objective of this study is to analyze the current visiting trends of patients with rosacea, and the prescription patterns of Korean dermatologists who are engaged in managing such patients.

## 2. Methods

### 2.1. Data Source

This retrospective cross-sectional study involved outpatients from 7 affiliated hospitals of The Catholic University of Korea. The data were extracted from the hospital database known as nU. This system contains information regarding all patients who visited one of the affiliated hospitals of The Catholic University of Korea, and thereby includes all data, regardless of national health insurance coverage. This study was reviewed and approved by the institutional review board of The Catholic University of Korea (XC19REDI0064), and was conducted according to the principles of the Declaration of Helsinki.

### 2.2. Study Population and Study Design

Our study included rosacea patients whose diagnosis was based on the International Classification of Diseases’ diagnostic codes for rosacea (L711, L718 or L719), and between 2007 and 2018, it was the primary diagnosis made by dermatologists from affiliated hospitals of The Catholic University of Korea. A total of 56,651 hospital records met the inclusion criteria. The primary outcomes of interest were the proportion of rosacea visits of all dermatology outpatient visits, and the prescription trends for rosacea over a twelve-year period. The secondary outcome measures included factors affecting systemic or topical prescriptions for rosacea. In addition, we examined the changes in the mean duration of systemic therapy over time.

### 2.3. Statistical Analysis

We performed the chi-square test to analyze significant differences between categorical variables. An independent t-test was performed to analyze the differences between numerical variables. If a record of a specific prescription was available on the day that the patient visited for rosacea, the patient was considered to have received a prescription for rosacea. The factors associated with each prescription were analyzed by conditional logistic regression analysis. A P-value of less than 0.05 was considered statistically significant. All statistical analysis was performed using IBM SPSS version 26.0 (IBM, Armonk, NY, USA).

## 3. Results

### 3.1. Patients’ Demographic Characteristics

Over the past 12 years, we identified a total of 56,651 visits for rosacea. The demographic characteristics of rosacea patients are summarized in Table 1. Overall, rosacea visits were most common in female patients aged between 40 to 59 years.

**Figure 1:**
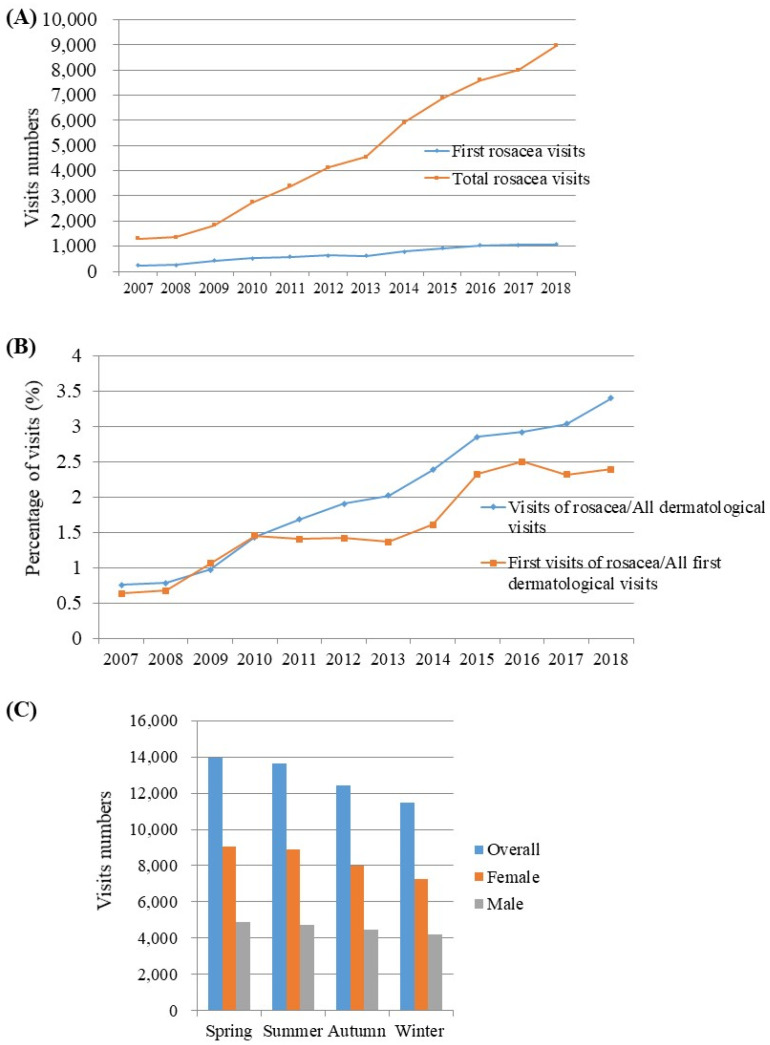
Time trends of the proportion of rosacea visits. (**A**) Number of total and first-time visits for rosacea between 2007 and 2018. (**B**) Temporal variation in the proportion of rosacea visits compared with all dermatological visits between 2007 and 2018. (**C**) The overall number of visits for rosacea in each season.

The annual number of rosacea visits to a dermatologist increased from 2456 during 2007–2012 to 6985 during 2013–2018. Our study found an increase in both the total number of rosacea visits and first-time rosacea visits in dermatology outpatient clinics (Figure 1). Of all dermatology outpatient visits, the visits for rosacea constituted 0.75% in 2007, which increased to 3.40% in 2018. Our study also revealed an increase in the proportion of first-time visits for rosacea out of all patients with dermatological ailments over the years. Patient visitation was most frequent in spring (27.08%), followed by summer (26.48%), autumn (24.14%), and winter (22.28%). However, there was no statistically significant difference in patient visitation between the seasons.

**Figure 2:**
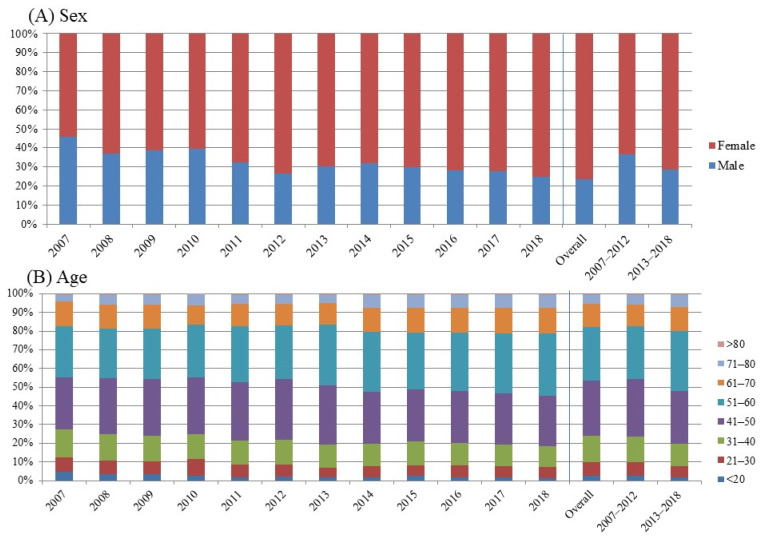
The distribution of rosacea visits by year of visit. (**A**) The distribution of rosacea visits by sex. (**B**) The distribution of rosacea visits by age.

The proportion of females presenting with rosacea was higher than that of males (Figure 2). However, there was no statistical difference in the annual visitation number between a female and male patient (*p* = 0.39). With regard to age, the proportion of those in their 40s and 50s was consistently high.

**Figure 3:**
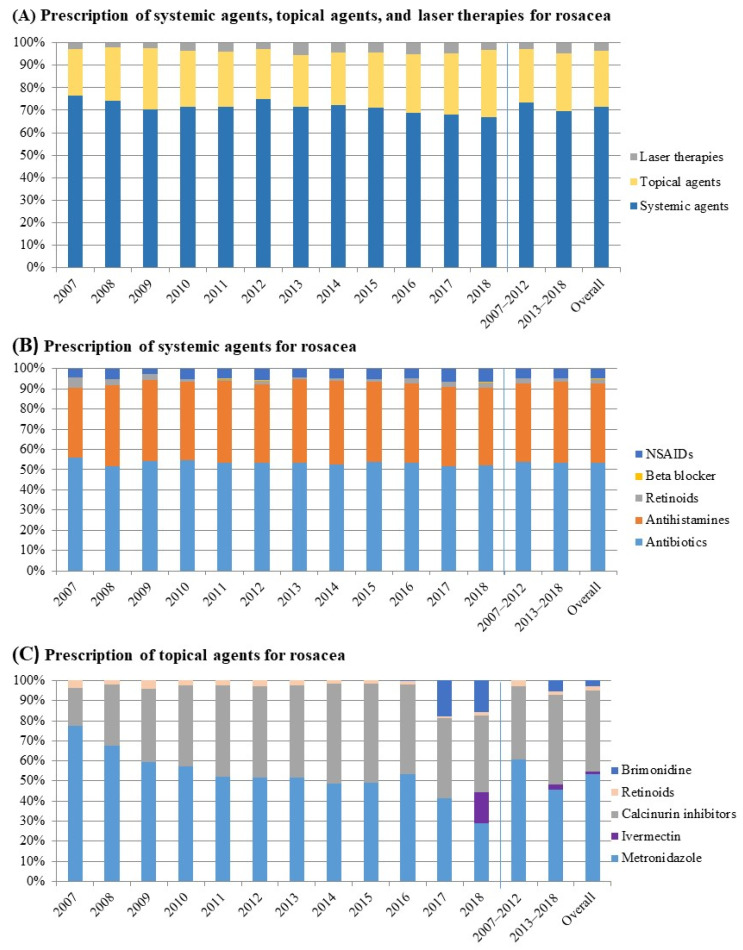
Trends in prescription patterns of rosacea. (**A**) Prescription of systemic and topical agents, and laser therapies for rosacea. (**B**) Prescription patterns of various systemic agents in rosacea. (**C**) Prescription patterns of various topical agents in rosacea.

To identify the changes in prescription trend for each treatment modality, we divided the entire observation period into two segments: the first six years (2007–2012) and the latter six years (2013–2018; Figure 3). The ratio between topical agents, systemic agents and laser therapy for rosacea remained constant between 2007–2012 and 2013–2018 (*p* = 0.77).

Among the systemic agents, no agent was particularly favored. Systemic antibiotics were the most prescribed, followed by antihistamines, non-steroidal anti-inflammatory drugs, and retinoids. The preferred topical agent for the treatment of rosacea changed between the periods 2007–2012 and 2013–2018 (*p* = 0.02). Metronidazole was the most commonly prescribed topical agent in 2007–2012, whereas calcineurin inhibitors became the top choice in 2013–2018. Of note, in Korea, topical brimonidine and ivermectin, which are newer, and are now key popular topicals, were introduced in 2017 and 2018, respectively.

**Figure 4:**
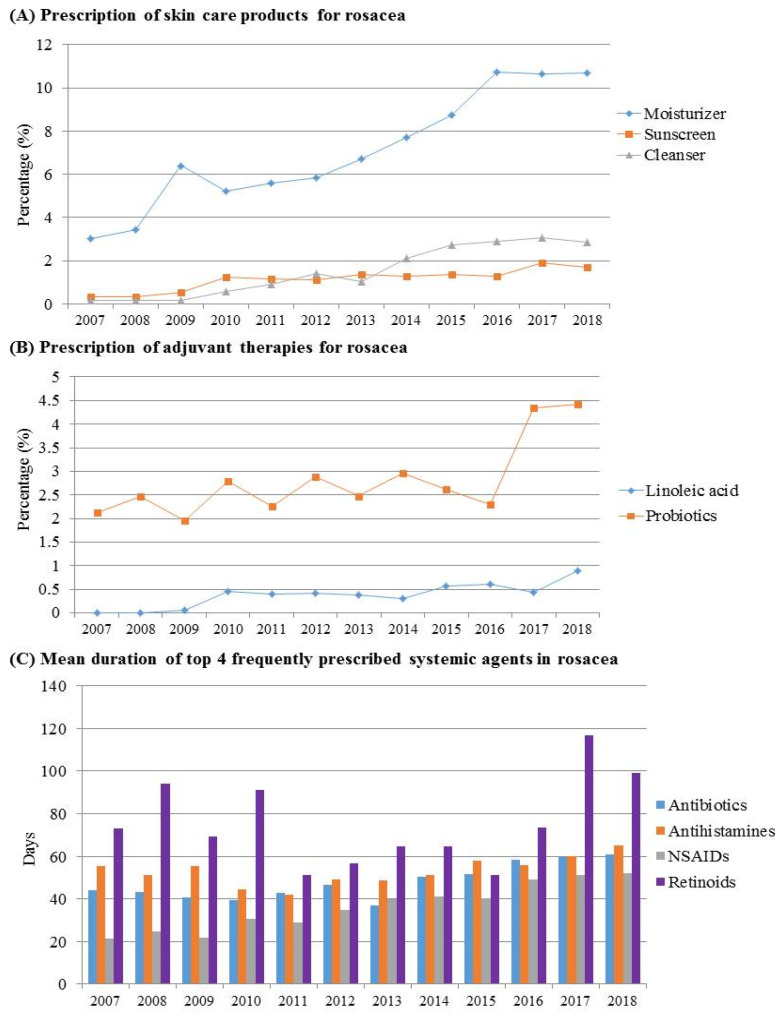
Prescription trends of skin care products and adjuvant therapies. (**A**) Prescription of skin-care products for rosacea. (**B**) Prescription of other adjuvant therapies for rosacea. (**C**) The mean duration of top 4 frequently prescribed systemic agents for rosacea.

We further analyzed the prescription pattern of skin care products by dermatologists (Figure 4). Skin care products such as sunscreens, moisturizers, and cleansers were prescribed to a rising proportion of rosacea patients, but not all. In addition, the prescription of oral probiotics as adjuvants by dermatologists increased over time, with a steep rise since 2017.

The duration of treatment with oral antibiotics increased gradually over time. To detect the changes in the duration of treatment with each systemic agent, we divided the entire observation period into two segments: the first six years (2007–2012) and the latter six years (2013–2018). There was a significant increase in the treatment duration with both oral antibiotics (*p* = 0.024) and NSAIDs (*p* < 0.001) between the two time periods. The average treatment duration with oral antibiotics from 2007 to 2012 was 42.75 days, which increased to 52.39 during 2013 to 2018. The treatment duration of oral NSAIDs increased from 27.02 days in 2007–2012 to 45.65 in 2013–2018.

### 3.2. Factors That Affect Dermatologists’ Prescriptions for Rosacea

Among a total of 56,651 visits for rosacea, more than one systemic agent was prescribed on 46,215 (81.57%) visits, and topical agents were prescribed in 41,436 (73.14%) visits. We further analyzed dermatologists’ prescription patterns for rosacea. Female patients showed increased odds of being prescribed with topical ivermectin (OR, 1.15; 95% CI, 1.02–1.31) and topical brimonidine (OR, 1.39; 95% CI, 1.22–1.58) compared with male patients with rosacea, whereas female patients showed decreased odds of receiving topical metronidazole (OR, 0.86; 95% CI, 0.82–0.91; Table 2). Increased odds of receiving a prescription for topical metronidazole (OR, 1.13; 95% CI, 1.25–1.38), CNIs (OR, 1.17; 95% CI, 1.11–1.23), or brimonidine (OR, 1.22; 95% CI, 1.08–1.37) were observed in rosacea patients over 50 years when compared with patients under 50, whereas decreased odds of a topical retinoid prescription (OR, 0.60; 95% CI, 0.47–0.77) were observed in patients older than 50 when compared with patients under 50.

Among systemic agents, females had decreased odds of receiving retinoids (OR, 0.48; 95% CI, 0.42–0.56) compared with male patients, while having increased odds of receiving systemic antibiotics (OR, 1.22; 95% CI, 1.21–1.27), antihistamines (OR, 1.23; 95% CI, 1.18–1.28), beta-blockers (OR, 2.57; 95% CI, 1.81–3.65), and NSAIDs (OR, 1.39; 95% CI, 1.27–1.51) compared with male patients.

Rosacea patients over 50 had increased odds of receiving systemic antihistamines (OR, 1.08; 95% CI, 1.04–1.12) and NSAIDs (OR, 1.13; 95% CI, 1.04–1.22), and decreased odds of receiving systemic antibiotics (OR, 0.84; 95% CI, 0.81–0.88) and retinoids (OR, 0.40; 95% CI, 0.34–0.47) for rosacea when compared with those under 50.

With regard to skin care products, the odds of dermatologists prescribing moisturizers (OR, 1.61; 95% CI, 1.51–1.72), sunscreens (OR, 2.20; 95% CI, 1.79–2.70), and cleansers (OR, 1.46; 95% CI, 1.27–1.68) were higher in female patients than in males, and the odds of prescribing cleansers (OR, 0.60; 95% CI, 0.53–0.69) were decreased in patients older than 50 years compared with patients under 50. In addition, laser therapy was more frequently performed on patients with rosacea who were aged under 50 years than in older patients (OR, 0.71; 95% CI, 0.66–0.77).

## 4. Discussion

In this study, the visitation trends of rosacea patients and the prescription patterns of dermatologists were reviewed over a 12-year period (2007–2018). In Korea, rosacea visits to dermatology outpatient clinics have increased over time.

The prevalence of rosacea varies widely across different studies. Based on the results of a systematic review and meta-analysis, the estimated prevalence of rosacea ranges between 0.00% and 23.14%, and the pooled proportion of patients with rosacea was 2.39% among dermatology outpatients [2]. Among the general population, the pooled proportion of patients with rosacea was 5.46% [2]. However, the prevalence of rosacea among the entire Korean population is not clearly established. As rosacea is not a subsidized disease in Korea, the prevalence of rosacea in the Korean population cannot be estimated accurately using the NHIC database. Although this EMR-based dataset does not represent the entire population, and may be an underestimate of the true number of rosacea patients, as this study includes an EMR dataset covering seven different medical centers located across Korea over a period of 12 years, we believe that our data represent the epidemiological characteristics of Korean patients with rosacea quite accurately. In this study, the increase in the frequency of rosacea visits over time reflects the rising prevalence of rosacea in Korea. A possible explanation for this increased visitation may be increased disease awareness in both physicians and patients [9,10].

In general, rosacea is more prevalent in individuals older than 30 years, especially those between their 30s and 50s [11,12]. Accordingly, in this study, rosacea was most prevalent in patients in their 40s and 50s. Although rosacea affects both sexes, a female preponderance was observed in our study, which is in line with the findings from recent studies [2,13]. The female predominance might be attributed to a greater self-awareness of their appearance and a desire for treatment, which leads to an increased number of hospital visits.

We did not observe any seasonality in the visiting trends for rosacea. Although the visits were expected to be the most frequent in summer as a result of stronger ultraviolet radiation, rosacea visits were most common in spring, followed by summer, and were the least frequent in winter. This is in line with the findings from Hancox et al. [14] where spring was the peak season for rosacea visits, constituting 27.5% of all annual visits, while the trough season was winter, which accounted for 23.0% of all annual visits. Increased sensitivity to ultraviolet radiation is commonly observed in the spring following a decrease in the protective pigment during winter, which may explain our findings to some degree. However, as this study only identified the hospital utilization pattern for rosacea, further studies are needed to confirm that this trend is directly associated with the course of the disease.

Overall, systemic agents were the most often prescribed, followed by topical agents and laser therapies. Among the topical agents, there was significant change in the favored agent. Whereas topical metronidazole gel was the mainstay of topical treatment for rosacea in the past, various topical agents have since been introduced and are being prescribed, which includes topical brimonidine and ivermectin. As the other novel topical agents for rosacea only became available very recently in Korea, topical calcineurin inhibitors were favored in Korea from 2013 to 2018. While Weissenbacher et al. [15] reported no superiority of topical pimecrolimus 1% cream over vehicle cream in the treatment of rosacea, an open-label randomized clinical study by Koca et al. [16] reported that topical pimecrolimus was effective in the treatment of papulopustular rosacea. We suggest that topical calcineurin inhibitors are relatively safe and effective in the long-term management of rosacea given their anti-inflammatory and immunomodulatory actions.

With regard to systemic agents, there was no significant change in prescription patterns. Overall, systemic antibiotics (52.45%) were most commonly prescribed, followed by antihistamines (39.38%), NSAIDs (4.94%), retinoids (2.12%), and beta-blockers (0.16%). The mean duration of treatment with oral antibiotics was 52 days during 2013–2018 and was increased by 10 days in 2007–2017. The treatment guidelines recommend 4–8 weeks of antibiotics for rosacea [17,18,19,20], and our overall course of antibiotic use for rosacea is in line with these guidelines. Although oral antibiotics are the treatment of choice for the papulopustular skin lesions of rosacea, long-term use of antibiotics can result in antibiotic resistance [21]. Therefore, clinicians should prescribe oral antibiotics more cautiously to achieve an optimal therapeutic response while minimizing adversity. Although the recent consensus guidelines for rosacea only recommend beta-blockers, doxycycline, and isotretinoin as systemic treatment modalities for rosacea [7], antihistamines and NSAIDs were frequently prescribed in real-world settings. Indeed, NSAIDs can attenuate the skin inflammation and angiogenesis in rosacea [22]. We suggest that this is probably due to their anti-inflammatory effect, and their ability to reduce the skin symptoms (i.e., pain and itch) that rosacea patients often complain of; therefore, antihistamines and NSAIDs are commonly prescribed for rosacea by dermatologists.

Systemic agents including oral antibiotics, antihistamines, and beta-blockers were prescribed more frequently in females than in males, which implies that females may be more sensitive to their skin condition. Retinoids were prescribed more frequently in males than in females, which could be explained by their effectiveness in reducing thickened skin (i.e., rhinophyma), which is more common in males [23].

The prescription of skin care products by dermatologists has increased over time, especially in females. Since general skin care is extremely important in managing rosacea [17], more emphasis should be placed on the use of proper skin care products, especially in males.

We found that there was a rise in the prescription of oral probiotics as an adjuvant therapy by dermatologists. Recent studies have found differences in the gut microbiome between patients with rosacea and healthy controls [24,25]. In addition, studies have shown that oral probiotics combined with conventional therapies are promising [26,27], which supports the prescription of oral probiotics for rosacea.

In conclusion, based on our study encompassing seven institutions across the country, dermatology outpatient visits by patients with rosacea are increasing in Korea. The real-world prescribing trends will help physicians provide better treatment strategies and will offer optimal guidance to patients.

## Figures and Tables

**Table 1:** Demographic characteristics of rosacea visits.

Variables	Rosacea Visits, n (%)
Age, year	
≤20	1117 (1.97)
21–30	3584 (6.33)
31–40	6946 (12.26)
41–50	16,301 (28.77)
51–60	17,614 (31.09)
61–70	7250 (12.80)
71–80	3476 (6.14)
>80	363 (0.64)
Sex	
Female	39,697 (70.07)
Male	16,954 (29.93)
Setting	
Metropolitan	25,000 (44.12)
Urban	31,651 (55.88)

**Table 2:** Factors affecting prescriptions for rosacea by dermatologists.

Variables	Sex (Case/Reference: Female/Male)			Age (years) (Case/Reference: ≥50/<50)		
	OR	95% CI	*p*-Value	OR	95% CI	*p*-Value
***Topical agents***						
Metronidazole	0.86	0.82–0.91	<0.001 *	1.13	1.25–1.38	<0.001 *
Ivermectin	1.14	1.02–1.31	0.02 *	1.10	0.98–1.24	0.09
CNIs	0.96	0.91–1.01	0.15	1.17	1.11–1.23	<0.001 *
Retinoid	0.81	0.64–1.03	0.09	0.60	0.47–0.77	<0.001 *
Brimonidine	1.39	1.22–1.58	<0.001 *	1.22	1.08–1.37	0.001 *
***Systemic agents***						
Antibiotics	1.22	1.21–1.27	<0.001 *	0.84	0.81–0.88	<0.001 *
Antihistamine	1.23	1.18–1.28	<0.001 *	1.08	1.04–1.12	<0.001 *
Retinoids	0.48	0.42–0.56	<0.001 *	0.40	0.34–0.47	<0.001 *
Beta blockers	2.57	1.81–3.65	<0.001 *	1.05	0.80–1.37	0.80
NSAIDs	1.39	1.27–1.51	<0.001 *	1.13	1.04–1.22	0.001 *
***Skin care products and laser therapy***						
Moisturizer	1.61	1.51–1.72	<0.001 *	0.95	0.89–1.00	0.07
Sunscreen	2.20	1.79–2.70	<0.001 *	0.96	0.82–1.14	0.71
Cleanser	1.46	1.27–1.68	<0.001 *	0.60	0.53–0.69	<0.001 *
Laser	1.00	0.92–1.08	0.99	0.71	0.66–0.77	<0.001 *
***Adjunctives***						
γ-linoleic acid	1.11	0.94–1.30	0.19	0.78	0.67–0.91	0.002 *
Probiotics	1.31	1.22–1.41	<0.001 *	0.96	0.90–1.03	0.34

Abbreviations: CI, confidence interval; CNI, calcineurin inhibitors; OR, odds ratio; NSAID, non-steroidal anti-inflammatory drug. * indicates a *p*-value less than 0.05.
